# A Combined Approach Using IncobotulinumtoxinA and CPM-Hyaluronic Acid Injections for the Periorbital Complex Improvement: A Pilot Study

**DOI:** 10.1093/asjof/ojae027

**Published:** 2024-05-22

**Authors:** Carla de Sanctis Pecora, Bruno Vella Pateo, Gisele Jacobino de Barros Nunes, Abdulay Ezequiel Machado Lanna Queiroz, Bruno Rebelo Lages da Silveira, Jani Van Loghen

## Abstract

**Background:**

Previous reports have objectively demonstrated the efficacy of botulinum toxin for brow elevation. No previous clinical trial has reported a combined approach to botulinum toxin A injection with hyaluronic acid (HA) injection in the upper face for periorbital region beautification focusing on eyebrow reshaping.

**Objectives:**

To evaluate the effect of IncobotulinumtoxinA injection using the ONE21 technique combined with HA injection (CPM technology) to redefine brow shape and position.

**Methods:**

A prospective pilot study was designed to evaluate the effect of IncobotulinumtoxinA injection using the ONE21 technique—with a preestablished scheme of doses and injection-site distribution—combined with HA injection (CPM technology) periosteally into the palpebromalar groove and subdermally in the anterior temporal region, to redefine brow shape and position. Objective eyebrow measurements were taken by an independent investigator using the Merz Aesthetic Scale (MAS) for brow positioning. Patient satisfaction was also evaluated. Some patients were also assessed using the Vectra System (Canfield Scientific, Parsippany, NJ).

**Results:**

Eleven females, aged 29 to 55 years, were included in this prospective pilot study. The totality of patients (11/100%) had at least ≥1-point improvement in the MAS brow positioning. All patients (100%) reported significant aesthetic improvement of their periorbital region and appearance, with 82% of the patients much improved. Mild side effects, such as ecchymosis and transient temporal edema, were reported.

**Conclusions:**

The combined technique improved the appearance of the orbital area by uplifting the lateral eyebrow and creating an almond-shaped eye effect, which characterizes the trending marketing term Foxy eyes. Further studies, including more cases, are needed to obtain a statistically significant outcome.

**Level of Evidence: 4:**

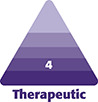

The aging process in the periorbicular region can start as early as the 1920s, because of soft-tissue ptosis, volume depletion, bone resorption,^1,2^ the action of the muscles of facial expression, clinically manifested as the appearance of orbital hooding, crow's feet, sunken eyes, infraorbital hollows,^3,4^ and loss and flattening of lateral eyebrow arches in older patients.^5,6^

Eyebrows have an important role in expressing emotions and in facial aesthetics.^7^ Their shape and positioning are directly related to the balance between the depressor muscles of the brow, which comprise the corrugator supercilii, procerus, depressor supercilii and orbicularis oculi muscles, and the single elevator muscle (ie, frontalis muscle), which inserts in the skin below the eyebrow, the area where its fibers interdigitate with the orbicularis oculi muscle.^8^ Moreover, the lateral eyebrow is less supported by deeper structures than the medial eyebrow, so the balance of forces acting on the eyebrow segments usually turns the lateral segment of the brow ptotic at earlier ages when compared with the medial segment.^9^

The current options to improve the eyebrow area are nonsurgical approaches, including toxins, lasers, microfocused ultrasound, threads, and fillers.^10^ The use of botulinum toxin is a popular option; however, the standard guideline includes treating brow depressors while keeping the frontalis muscle free of injection in order to avoid eyebrow and eyelid dropping.

For more youthful look and a lifting effect on the eyebrows, one should temper the balance between the frontalis muscle, the only elevator in the upper face, and the eyebrow depressors, weakening the medial and lateral depressor muscles.^8^ Some investigators have suggested that the desired female eyebrow peak has been moving more laterally over the last 100 years.^11^ In the beginning of 20th century, a thin and arched eyebrow with the peak coincident with the mid-pupillary line (MPL) was considered the ideal beauty pattern; currently, the ideal beauty pattern is a more flattened, thicker eyebrow, with a lateral arching effect coincident with the outer canthus.^11^ Nevertheless, as there is no one-size-fits-all concerning the ideal eyebrow position, and considering gender and ethnic anatomic particularities, we understand that an individualized treatment is the best option to reach the best aesthetic result and patient satisfaction. The objective of this study is to describe a combined technique designed to beautify of the periorbital complex by uplifting the lateral eyebrow and giving the eye an almond shape; this current trend is called “Foxy eyes” (Figure 1).^12^

**Figure 1.**
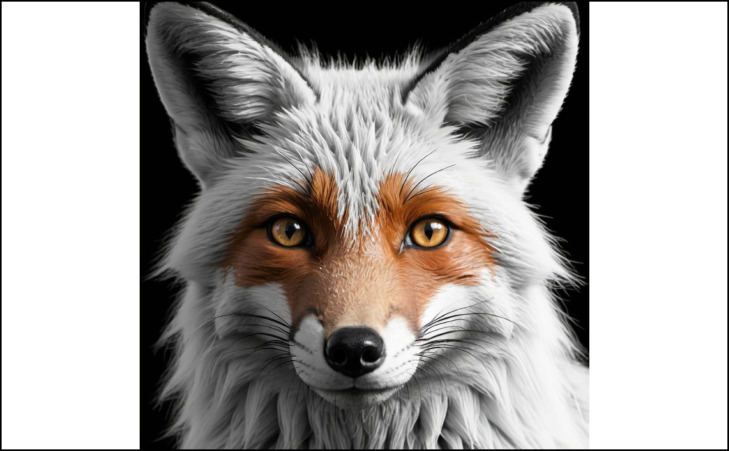
The Foxy eye. This trend was inspired in the Fox eye shape, an almond-shaped eye with the lateral canthus placed uppermost compared with the inner canthus. The laterally arched eyebrow shape was included in the technique to create an optical illusion and corroborate the perception of almond-shaped eyes.

## METHODS

All procedures performed in this report involving human patients were in accordance with the ethical standards of the institutional and/or national research committee and with the 1964 Declaration of Helsinki and its later amendments or comparable ethical standards.

### Ethics Approval

The report was approved by a centralized institutional review board (Hospital e Centro de Reabilitação da Associação de Assistência à Criança Deficiente number: 74950323.8.00 00.0085, approval date: November 6, 2023). Written informed consent has been provided by all the patients to have the case details and any accompanying images published.

### Design

This is a single-center prospective study conducted at the Dermatologie Clinica, located in the city of São Paulo, Brazil. Eleven 29- to 55-year-old healthy female patients were enrolled in this pilot study from July to December 2020. An informed consent form was provided and signed by all patients. Exclusion criteria were pregnancy, breastfeeding, history of autoimmune disease, history of rheumatic fever, marked facial asymmetry, known hypersensitivity to the study medication, and any facial treatment with botulinum toxin, permanent or biodegradable fillers within the last 12 months.

**Figure 2.**
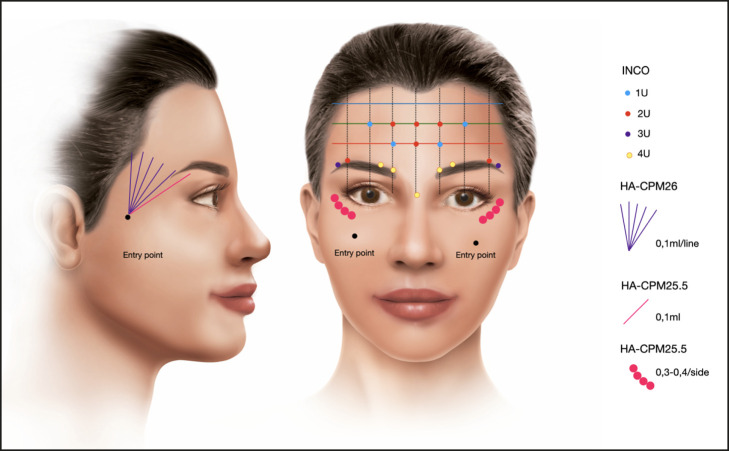
Treatment scheme. Dose and injection-site distribution scheme of IncobotulinumtoxinA: 12 U forehead, 20 U glabella (5 sites with 4 U), 5 U/side orbicularis oculi. CPM-HA25.5 injection at the periosteal level in the palpebromalar groove, and CPM-HA26 injection in the subdermal plane of the anterior temporal region.

The study evaluated the effect of a holistic technique on brow shape and positioning by injecting IncobotulinumtoxinA (INCO; Xeomin, Merz Pharmaceuticals GmbH, Frankfurt, Germany) using the ONE21 technique (Figure 2)^13^ in the upper face combined with cohesive, polydensified, monophasic hyaluronic acid of 25.5 mg/mL (CPM-HA25.5; Belotero Intense; cohesive polydensified matrix HA; Merz Pharmaceuticals GmbH) gel injected into the palpebromalar groove. In 5 of the 11 patients, the anterior temporal region was clinically deflated and was also treated by injecting cohesive polydensified matrix HA of 26 mg/mL (CPM-HA26; Belotero Volume Merz Pharmaceuticals GmbH) into the subdermal superficial fat layer of the anterior temporal region.

It was a single visit protocol performed starting with botulinum toxin injection (the first step), followed by the injection of the CPM-HA25.5 into the palpebromalar groove (the second step), and just 5 patients were submitted to a third step—CPM-HA26 injection into the anterior temporal region. On baseline and Day 14, pictures were taken, and patients completed a 7-point questionnaire based on Global Aesthetic Improvement Scale. The photographs taken with standardized distance, light with frontal and oblique views at the following time points: D0 and D14 and were evaluated by an independent, blinded, specialized observer who was asked to classify patients using the Brow Positioning Merz Aesthetic Scale (MAS).^14^ Vectra System H1 (Canfield Scientific, Parsippany, NJ) Vector displacement quantitative analysis was performed to evaluate eyebrow displacement at D14. The smallest and the greatest displacement measurements were considered for each patient.

### Injection Technique

The 100 U vial of INCO (Xeomin, Merz Pharmaceuticals GmbH) was reconstituted by adding 2 mL of sterile saline solution. Thirty gauge needles and 1 mL syringes were used for injection. All patients were injected using the ONE21 technique^13^ into the forehead, glabella, and periorbital regions, using a predetermined scheme of injection point distribution and dosage, as shown in Figure 2. IncobotulinumtoxinA was injected into the medial and lateral brow depressors, with 20 U on the glabella and 2 points of 2 and 3 U in the superior lateral fibers of the orbicularis oculi, in the inferior border of the eyebrow respecting 1 cm from the orbital rim; combined with the injection of the inferior and medial central portions of the frontalis muscle, whereas the inferior lateral portion of the forehead remained untreated, increasing the resting tonus of the latter, creating a predictable lateral arching lifting effect of the eyebrow.

In the same session, 0.4 mL per side of CPM-HA25.5 was injected into the palpebromalar groove using a 25G, 38 mm nontraumatic cannula (TSK Steriglide, Japan) and a retrograde linear threading technique. The entry point was placed in the anterior malar area, 3 to 4 cm below the inferior orbital rim, on the MPL (Video 1). The cannula was advanced upward and laterally, crossing the zygomatico-cutaneous retaining ligament.^15^ Small aliquots of CPM-HA25.5 were delivered along the lateral border of the orbital rim, at the periostea level, below the orbicularis-retaining ligament (ORL). The product was distributed from 0.2 to 0.3 cm above the lateral canthus and stopped 1 cm lateral to a line passing through the MPL. On the superior lateral orbital rim, immediately below the eyebrow and retro-orbicularis fat, 0.1 mL of CPM-HA25.5 was delivered at the supraperiosteal plane to enhance further support for the eyebrow (Figure 2).

In the third step of the protocol, with an entry point at the mid-portion of the zygomatic arch, a 25G, 50 mm blunt tipped cannula was advanced upward and medially, using a retrograde linear threading technique, injecting small amounts in multiple passes totaling approximately 0.5 mL of CPM-HA26 per side into the subdermal space of the anterior temporal region, between the hairline and the lateral orbital rim (Figure 2). Injection technique of second and third step can be seen in Video 1. When injecting in the temporal area, it is important to be aware of anatomy. In this region, arteries and veins are located in superficial layers of tissue as well as in the deeper layers. The superficial temporal artery, a branch of the external carotid artery, is located inside the superficial temporal fascia and has connections to the supraorbital artery which is a branch of the ophthalmic artery. In order to be safe, one should stay strictly hypodermal.^16^

## RESULTS

A total of 11 females were enrolled in this study, aged 29 to 55 years of age. Most of them (9/11) were Caucasian, 1 was of African descent, and 1 was of Asian descent. Nine out of 11 (82%) had previously undergone treatment with botulinum toxin for glabellar and frontal wrinkles over 6 months earlier. An equal number of patients (9) had already received facial treatment with dermal fillers previously, but not in the same area as in this study. Patients 1 to 5 were treated with the 3 steps of the treatment (incobotulinumtoxin injection, injection of the CPM-HA25.5 in the palpebromalar hollow, and injection of the CPM-HA26 in the anterior temporal region); and Patients 6 and 7 only with 2 steps (incobotulinumtoxin injection and injection of the CPM-HA25.5 in the palpebromalar hollow).

**Figure 3.**
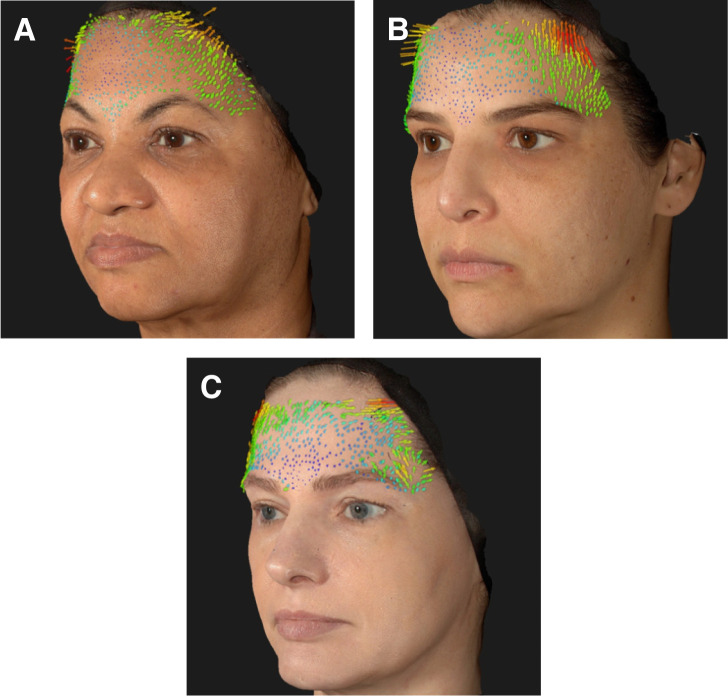
Vectra System H1 Vector displacement quantitative analysis of the upper face, demonstrating the lateral lifting effect of the eyebrow after the 3-step treatment, showing arrows directing upwards in (A) a 54-year-old female patient, (B) a 39-year-old female patient, and (C) a 45-year-old female patient. Vector arrows provide a precise indication of the direction and magnitude of skin movement while comparing the 15 days after treatment pictures with baseline. The colors, size, and direction of the arrows are related to the lifting intensity. Lifting intensity increases from blue, green, yellow, orange to red, and also with the length of the arrow. The greater is the lifting the larger the arrow will be.

**Figure 4.**
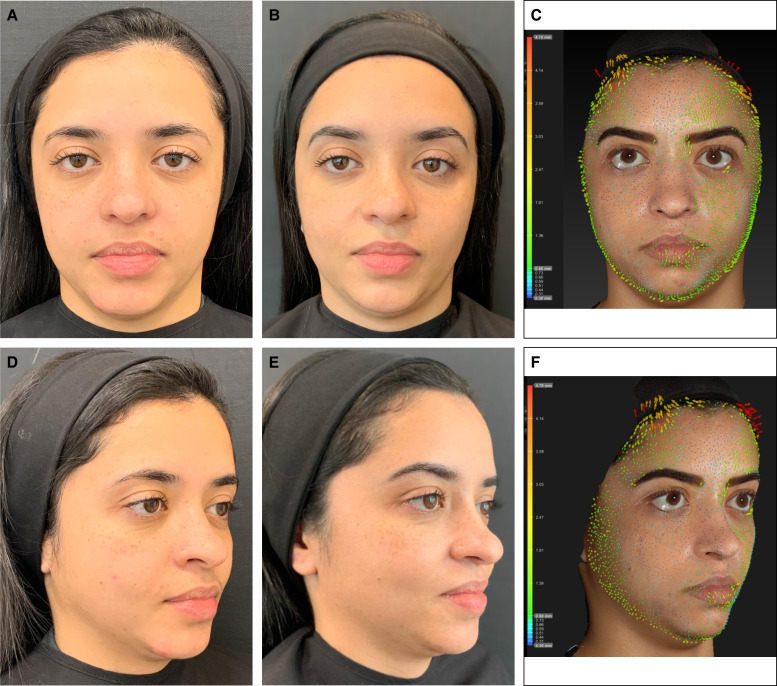
A 29-year-old female treated with the 2-step protocol (45 U of incobotulinumtoxin and injection of the CPM-HA25.5 in the palpebromalar hollow). (A, D) Before treatment, (B, E) 15 days after treatment with a 2-point improvement in the brow shape and positioning (Merz Aesthetic Scale), and (C, F) Vectra System H1 Vector displacement quantitative analysis, demonstrating arrows directed upward in the lateral part of the eyebrows. Lifting intensity increases from blue, green, yellow, orange to red.

**Figure 5.**
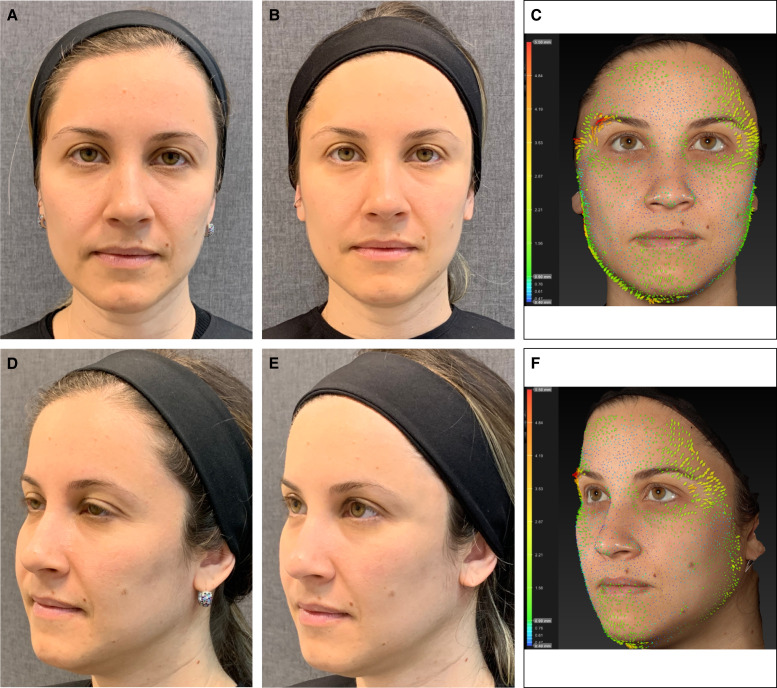
A 35-year-old female treated with the 3-step protocol (45 U of incobotulinumtoxin, injection of the CPM-HA25.5 in the palpebromalar hollow and injection of the CPM-HA26 in the anterior temporal region). (A, D) Before treatment, (B, E) 15 days after treatment with a 1-point improvement in the brow shape and positioning (Merz Aesthetic Scale), and (C, F) Vectra System H1 Vector displacement quantitative analysis, demonstrating arrows directed upward in the lateral part of the eyebrows. Lifting intensity increases from blue, green, yellow, orange to red.

**Figure 6.**
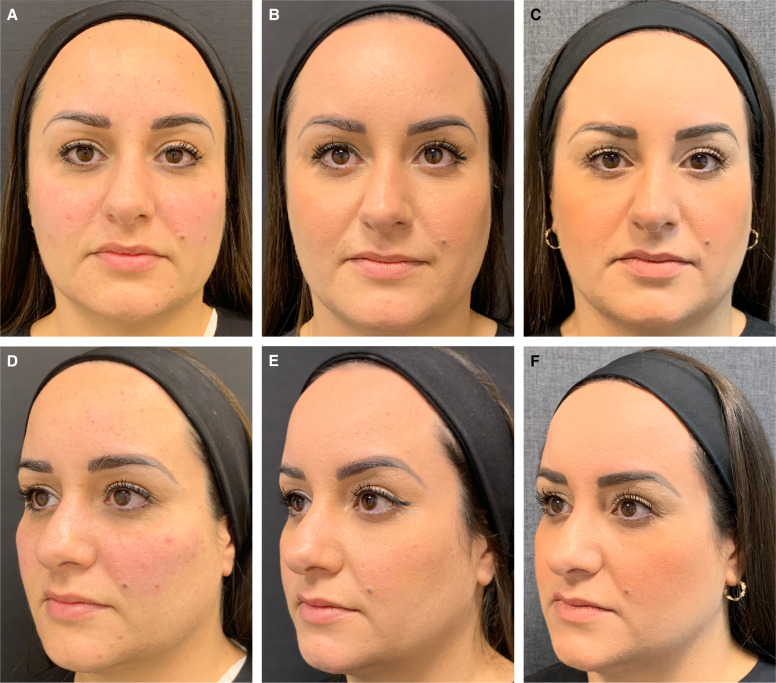
A 35-year-old female treated with the 2-step protocol (45 U of incobotulinumtoxin and injection of the CPM-HA25.5 in the palpebromalar hollow). (A, D) Before treatment, (B, E) 15 days after treatment, and (C, F) 5 months after treatment. Notice the arching effect achieved 15 days after treatment (a 3-point improvement in the Merz Aesthetic Scale for eyebrow shape and positioning). After 5 months, the patient still presented an arching effect compared with the before picture; however, less important when compared with the 15 days after treatment picture, demonstrating that the combination of the neurotoxin injection with filler injection is a key to achieving the technique full effect.

Nine patients (82%) reported significant aesthetic improvement of their periorbital region and appearance and classified the treatment with the highest score of the scale (7, much improved—Table 1, Figures 3-6), and 2 (18%) moderate improvement. All 11 patients (100%) involved in this pilot study had at least 1-point improvement in the MAS brow positioning (Table 2). Vectra System H1 Vector displacement quantitative analysis was performed in 7 out of the 11 patients. Not all patients had pretreatment Vectra images, so the analysis could not be performed for all patients. Nonetheless, all 7 evaluated patients presented a lateral eyebrow lift displacement of at least 3.6 mm in the greatest measurement (Table 3). Patients were followed up for 5 months, presenting a partial effect of the lateral arching effect of the eyebrow once the neurotoxin blocking effect faded out. No serious adverse events were reported, with most adverse events deemed mild and transient in nature as injection-site ecchymosis.

**Table 1.** Independent Investigator Assessment of the Brow Shape and Positioning.

	Score before treatment	Score 15 days after treatment
Patient 1	3	2
Patient 2	4	3
Patient 3	3	3
Patient 4	3	2
Patient 5	4	3
Patient 6	2	1
Patient 7	3	1
Patient 8	2	0
Patient 9	2	2
Patient 10	2	0
Patient 11	3	1

Patients 1 to 5 were treated with the 3 steps of the treatment (incobotulinumtoxin injection, injection of the CPM-HA25.5 in the palpebromalar hollow, and injection of the CPM-HA26 in the anterior temporal region), whereas Patients 6 to 11 were treated with the 2 steps (incobotulinumtoxin injection and injection of the CPM-HA25.5 in the palpebromalar hollow).

**Table 2.** Lateral Arching of the Eyebrows: GAIS Questionnaire—Subjective Assessment

GAIS	Much improved	Moderate improved	Minimally improved	No change	Minimally worse	Moderate worse	Much worse
Lateral arching of the eyebrows	81.81	18.18	0	0	0	0	0

GAIS, Global Aesthetic Improvement Scale.

**Table 3.** Vectra System H1 Vector displacement quantitative analysis focusing on the lateral part of the eyebrows

	Lateral eyebrow lifting smallest displacement (mm)	Lateral eyebrow lifting greatest displacement (mm)
Patient 1	0.9	5.5
Patient 2	1.4	11
Patient 3	1.1	8.4
Patient 4	1.6	9.6
Patient 5	2.4	6.8
Patient 6	0.3	3.6
Patient 7	0.8	4.7

Vectra System H1 Vector displacement quantitative analysis was performed in 7 of the 11 patients treated, focusing on the lateral part of the eyebrows. The displacement range is displayed and comprises smallest displacement (the green arrows) and greatest displacement (red arrows) observed in each case. Lifting intensity increases from blue, green, yellow, orange to red.

## DISCUSSION

For both facial expression and beauty, the eyebrow plays a central role has been and cosmetic concern since Ancient Egypt, when ink was used to paint eyelashes and eyebrows to highlight the region of the eyes.^8,17^

Eyebrows are one of the main features that determine upper facial beauty, known as the face’s mainline, being used as a reference for all other angles and contours of the face. There is great variety in shape, position, and size of the brows, which can be influenced by various factors, including age, sex, culture, ethnicity, and current fashion trends.^17^ For instance, in females, the eyebrow tends to have a pleasant arch peaking in the lateral third of the eyebrow and, furthermore, a club-shaped medial portion, whereas the male brow is flatter and fuller, and runs over the orbital rim without peaking and arching.^17^

With aging, the eyebrow tends to lose its lateral arch, having a flattened appearance because of the increase of the orbital aperture and width, bone atrophy, loss of the fat pads (in the forehead and temporal regions), decrease of the skin collagen content, as well as reduction of the tonus of the frontalis muscle. Nevertheless, a paradoxical elevation of the brow, in particular in medial and mid brow, can occur because of chronic activation of the frontalis muscle to overcome the levator system's weakness and/or the presence of redundant upper lid skin.^18^ Moreover, the eyebrow shape and position must be aesthetically pleasing on each particular face, taking into account the balance with other facial features.^19^ In the last century, the eyebrow beauty pattern changed considerably, with the eyebrow peak positioning moving from the MPL to the lateral canthus line, with the lateral end of the brow placed higher when compared with the medial end, providing a lateral arching effect. For instance, for cosmetic rejuvenation of the periorbital area, some authors suggest to restore the positioning of the apex the lateral two-thirds of the eyebrow.^20^ Currently, the trend is a lateral arched eyebrow, with the tail positioned even higher, associated with almond-shaped eyes, which is referred to as Foxy eye.

The effectiveness of botulinum toxin for lifting and changing the shape of eyebrows has been previously demonstrated.^21^ As eyebrow positioning is determined by the balance among the frontalis, responsible for its elevation, and the medial and lateral brow depressor muscles, to create a lifting effect of the eyebrows using neurotoxin, the medial and lateral brow depressors should be injected, sparing the frontalis. Furthermore, eyebrow positioning may also change after injection of botulinum toxin A (BoNT-A) into the glabella, as a result of its diffusion into the medial fibers of the frontalis, leading to an increased resting tonus in the remainder of the muscle and elevation of the eyebrow position.^22^

In the current protocol, IncobotulinumtoxinA was injected into the medial and lateral brow depressors combined with the injection of the inferior and medial central portion of the frontalis muscle, whereas the inferior lateral portion of the forehead remained untreated, increasing the resting tonus of the latter, creating a predictable lateral arching lifting effect of the eyebrow. The use of a botulinum toxin with a predictable halo of action (clinical area of effect^23^) provides the precision required for a more accurate treatment, preventing any undesirable results.

The filling of the lateral inferior region of the orbit, just below the orbital retaining ligament, with HA microboluses of CPM-HA25.5 places the ORL into a more horizontal orientation, provides regional structure support of the lateral border of the inferior eyelid lifting the corner of the eye with an almond-shaped eye effect, and increases the lateral lifting vector, rendering a quantifiable lifting effect of the treated area (Table 3).^15^ Injections in the superficial anterior temporal region, lateral to the line of ligaments, an imaginary line formed by true osteocutaneous ligaments, extending from the temporal crest to the mandible,^24^ generates a lifting effect, and particularly when injected in the superior compartment, separated by the inferior temporal septum, results in a lateral eyebrow lifting (Figures 3-6).^15^ Moreover, the technique has better outcomes if performed with the use of an HA with high F_N_ (normal force; provides the product with high resistance to static compression and reflects the ability of the product to maintain tissue projection), high viscosity (ability of the product to remain in the injected place), high elastic moduli *G*′ and *E*′ (related to capacity to withstand the mechanical stress throughout the dynamic expression of the face), and high cohesivity (related to better tissue integration).^25^

To our knowledge, this is the first study to describe a combined approach for periorbital region beautification focusing on eyebrow reshaping with the use of BoNT-A and HA injection. The present preliminary study demonstrates the achievement of a Foxy eye effect with the use of botulinum toxin and HA injection into the inferolateral edge of the orbit, and subdermally in the anterior temporal region. This beautifying procedure using the Foxy eye protocol promotes lifting and lateralization of the eyebrow tail with its lateral end placed superiorly compared with the medial end and an almond-shaped eye effect, with a high level of patient satisfaction. Our results highlight the importance of the assessment and treatment of the periorbital region as a unit. To achieve the so called Foxy eyes effect, it was necessary to combine more than one approach once the anatomical components of the periorbital area are closely related and play an important role in the eyebrow shape and positioning. In the current technique, we can observe that the frontalis muscle treatment not only can lead to an eyebrow lift, but that it also has an important role in obtaining the almond eye effect. Matsushita et al demonstrated the influence of the eyebrow shape on the perception of eye positioning, being the eyes perceived to be somewhat inclined in the same direction as the eyebrows.^12^ Thus, the laterally arched eyebrow shape was included in the technique to create an optical illusion and corroborate the perception of almond-shaped eyes.

Of the 11 patients involved in the study, 82% (9) reported significant improvement in the periocular region, and 18% (2) moderate improvement, with at least 1 point of eyebrow positioning improvement in the MAS, as analyzed by an independent, blinded evaluator. Further studies with larger sample size and longer follow-up are needed to validate the results presented herein. Among the weaknesses of our study, we highlight the small sample size, lack of Vectra analysis for all patients, limited diversity, a single-blinded observer, lack of control, different treatment approaches for half of the patients, and a short period of FU.

## CONCLUSIONS

The technique using IncobotulinumtoxinA injection into the forehead and brow depressors combined with CPM-HA injection into the palpebromalar groove and the anterior temporal region showed to be effective in creating the Foxy eye effect, characterized by a lateral arching effect on the eyebrow, with a lateral orbital support and almond-shaped eyes. Further studies, including more cases, are needed to obtain a statistically significant outcome.

## Supplementary Material

ojae027_Supplementary_Data
